# Supplementary material for: Magnitude and Extent of Helicoverpa zea Resistance Levels to Cry1Ac and Cry2Ab2 across the Southeastern USA
Source: Insects. 2023 Mar 7;14(3):262. doi: 10.3390/insects14030262 (PMC10058025; doi:10.3390/insects14030262)
Supplement: Supplementary file 1 [file insects-14-00262-s001.zip › insects-2158602-supplementary.pdf]

Table S1. Source and fate of *H. zea* collections.

Year	State	County or City	GPS	Collection Date	Host	Date Bioassay Performed, *H. zea* generation		Reason Bioassay(s) not Performed	
						Cry1Ac	Cry2Ab2 (2019 only)	Cry1Ac	Cry2Ab2
2018	AL	Augusta	32.4379, -86.5982	9 July	non-Bt corn	-	-	F_1 females laid non-viable eggs	-
2018	AL	Montgomery	32.4036, -86.1778	20 June	non-Bt corn	-	-	F_1 females laid non-viable eggs	-
2018	FL	Santa Rosa	30.7747, -87.1367	29 June	VT2PRO[a] corn	-	-	F_1 females laid non-viable eggs	-
2018	GA	Sumter	32.0378, -84.3696	18 July	non-Bt corn	25 Sept., F_2[b]	-	-	-
2018	GA	Tifton	31.3913. -83.5484	23 July	non-Bt sweet corn[c]	-	-	-	-
2018	NC	Lenoir	35.2436, -77.7748	2 July	non-Bt corn	5 Aug., F_1	-	-	-
2018	NC	Sampson	35.2591, -78.3564	25 June	non-Bt corn	5 Aug., F_1	-	-	-
2018	SC	Barnwell	33.3504, -81.3064	18 June	non-Bt corn	24 July, F_1	-	-	-
2018	SC	Barnwell	-	5 Aug.	grain sorghum[d]	-	-	Lost colony during hurricane	-
2018	SC	Darlington	34.2979, -79.7398	5 July	VT2PRO corn	-	-	F_1 females laid non-viable eggs	-
2018	SC	Darlington	34.2979, -79.7398	12 July	non-Bt corn	-	-	Not enough viable eggs	-
2018	VA	Suffolk	36.5826, -76.5966	12 July	VT2PRO corn	-	-	Not enough viable eggs	-
2018	VA	Suffolk	36.6774, -76.7556	20 July	non-Bt corn	-	-	Not enough viable eggs	-
2018	VA	Suffolk	36.5827, -76.5966	20 July	non-Bt corn	-	-	Not enough viable eggs	-

2018	VA	Dinwiddie	37.1312, -77.8951	17 Aug.	unknown	-	-	Not enough viable eggs	-
2019	AL	Autagua	32.4402, -86.5948	16 June	Herculex[e] corn	-	-	Not enough viable eggs	Not enough viable eggs
2019	AL	Henry	31.3591, -85.3205	25 June	non-Bt corn	2 Aug., F_1	31 July, F_1	-	-
2019	AL	Macon	32.4235, -85.8402	8 July	non-Bt corn	-	-	unknown	unknown
2019	FL	Santa Rosa 1[f]	30.7759, -87.1365	21 June	Obsession[a] sweet corn	31 July, F_1	2 Aug., F_1		
2019	FL	Santa Rosa 2	30.7759, -87.1365	17 June	Bt and non-Bt corn	-	-	unknown	unknown
2019	NC	Washington 1	35.8523, -76.6518	unknown	single female from a light trap	17 July, F_1	18 July, F_1	-	-
2019	NC	Washington 2	35.8523, -76.6518	unknown	single female from a light trap	17 July, F_1	19 July, F_1	-	-
2019	NC	Edgecombe	35.9905, -77.7619	unknown	non-Bt corn	unknown	unknown	-	-
2019	NC	Surry	36.4761, -80.6408	unknown	non-Bt corn	28 Oct., F_2	28 Oct., F_2	-	-
2019	NC	Wayne	35.2792, -77.8558	unknown	non-Bt corn	15 Oct., F_2	15 Oct., F_2	-	-
2019	NC	Wilkes	36.3162, -80.992	unknown	non-Bt corn	1 Oct., F_2	2 Oct., F_2	-	-
2019	SC	Barnwell 1	33.3516, -81.3073	2 July	non-Bt corn	29 July, F_1	29 July, F_1	-	-
2019	SC	Barnwell 2	33.3152, -81.3073	2 July	non-Bt corn	5 Aug., F_1	unknown	-	-
2019	SC	Darlington	34.2979, -79.7398	1 July	non-Bt corn	unknown, F_2	unknown, F_3	-	-
2019	VA	Suffolk	36.6787, -76.7548	8 July	non-Bt corn	-	5 Aug, F_1	unknown	-

[a]Bayer Crop Science. [b]Indicates the generation that we bioassayed. [c]*Zea mays* convar. *saccharata* var. *rugosa*. [d]*Sorghum bicolor*. [e]Corteva, Indianapolis, ID. [f]Denotes that we made multiple collections within the same county. Refer to the GPS coordinates for the specific location within the same county.
